# Pareidolia in Parkinson's Disease and Multiple System Atrophy

**DOI:** 10.1155/2021/2704755

**Published:** 2021-10-31

**Authors:** Kentaro Kurumada, Atsuhiko Sugiyama, Shigeki Hirano, Tatsuya Yamamoto, Yoshitaka Yamanaka, Nobuyuki Araki, Masatsugu Yakiyama, Miki Yoshitake, Satoshi Kuwabara

**Affiliations:** ^1^Department of Neurology, Graduate School of Medicine, Chiba University, Chiba, Japan; ^2^Medical Center for Dementia, Chiba University Hospital, Chiba, Japan; ^3^Department of Rehabilitation, Division of Occupational Therapy, Chiba Prefectural University of Health Sciences, Chiba, Japan; ^4^Urayasu Rehabilitation Education Center, Chiba University Hospital, Urayasu, Japan

## Abstract

Pareidolia is a visual illusion of meaningful objects that arise from ambiguous forms embedded in visual scenes. Previous studies showed that pareidolias are frequently observed in patients with Parkinson's disease (PD) as well as dementia with Lewy bodies. However, whether pareidolias are useful for differentiating PD from other neurodegenerative parkinsonism disorders including multiple system atrophy (MSA) is unclear. The noise pareidolia test (NPT) was performed in 40 and 48 patients with PD and MSA, respectively. A receiver operating characteristic (ROC) curve analysis was used to evaluate sensitivity and specificity. Results of neuropsychological tests were also compared between patients with PD with and without pareidolias. Visual hallucinations were present in none of the subjects. Pareidolic response in the NPT was observed in 47.5% and 18.8% of patients with PD and MSA, respectively. The number of pareidolic responses in patients with PD was significantly larger compared with patients with MSA (*P*=0.001). ROC curve analyses showed the sensitivity and specificity at 33% and 98%, respectively. Among patients with PD, those with pareidolias demonstrated higher State-Trait Anxiety Inventory-state (*P*=0.044) and State-Trait Anxiety Inventory-trait (*P*=0.044) than those without pareidolias. Pareidolias can be found in patients with PD without visual hallucinations, and the pareidolia test may be a highly specific test for differentiating PD from MSA. Thus, anxiety may be associated with pareidolias in patients with PD.

## 1. Introduction

Visual symptoms are common in Parkinson's disease (PD), which include double vision, blurry vision, watery eyes, illusions, feelings of presence and passage, and visual hallucinations [1, 2]. Moreover, visual illusions have been categorized as minor hallucinations [1] and have been reported to be present in 4.2%–19% of patients with PD [1, 3, 4].

Pareidolias are visual illusions of meaningful objects that arise from ambiguous forms embedded in visual scenes and have phenomenological similarities with visual hallucinations [5]. The pareidolia test has been reported as a tool for evoking and measuring pareidolias with good test-retest/inter-rater reliability [5–7]. The number of illusory responses in the pareidolia test was significantly correlated with the severity of visual hallucinations, suggesting the usefulness of the pareidolia test as a surrogate marker of visual hallucination in dementia with Lewy bodies (DLB) [5–7]. Moreover, the pareidolia test exhibited high diagnostic accuracy for discriminating DLB from Alzheimer's disease [5, 7]. In patients with PD, a previous study using the pareidolia test and positron emission tomography showed that pareidolias were more frequently found in healthy controls and associated with hypometabolism in the parietal cortex [8]. However, neural mechanisms of pareidolias in patients with PD are not fully understood. Whether pareidolias evoked by the pareidolia test are useful in differentiating PD from other neurodegenerative parkinsonism disorders including multiple system atrophy (MSA) is not yet clear.

The present study aimed to evaluate the usefulness of pareidolias evoked by the pareidolia test for differentiating patients with PD from MSA. Additionally, the clinical background and results of neuropsychological tests between patients with PD with and without pareidolias were compared to explore the underlying neural mechanism of pareidolias in patients with PD.

## 2. Materials and Methods

### 2.1. Subjects

This retrospective study was approved by the institutional review board of Chiba University Hospital. Moreover, informed consent was waived.

Patients with PD and MSA who underwent the pareidolia test between May 2018 and October 2020 at the institution of the current study were identified from the database. The pareidolia test was ordered by the treating physician for patients with different disease stages for initial evaluation or for indicating device-aided therapy in about one-fifth of the patients with PD. On the contrary, almost all patients with MSA underwent the test, except for those who could not due to visual loss or other reasons. The inclusion criteria for PD and MSA were patients who qualified the UK Parkinson's Disease Society Brain Bank Diagnostic Criteria [9] and the criteria described in the second consensus statement by Gilman et al. [10], respectively. The exclusion criteria were [1] current or history of another neuropsychiatric disorder and [2] taking antipsychotics, anticholinesterase inhibitor, or *N*-methyl-D-aspartate receptor antagonist. Based on these criteria, 40 and 48 patients with PD and MSA were included in the present study, respectively. MSA diagnosis was classified according to whether the clinical syndrome was dominated by parkinsonism (MSA-P) or cerebellar ataxia (MSA-C). Among the 48 patients with MSA, 19 and 29 were classified as having MSA-P and MSA-C, respectively. The diagnoses for all subjects remained stable during a median 10.5 months (range: 1–37 months) of clinical follow-up after the pareidolia test.

The medical records of both patients with PD and MSA were reviewed for age and disease duration at the pareidolia test, Frontal Assessment Battery (FAB), and Addenbrooke's Cognitive Examination (ACE-III) [11]. For patients with PD, detailed clinical profiles and results of the neuropsychological test including State-Trait Anxiety Inventory (STAI) [12], Hamilton Rating Scale for Depression (HAMD-17) [13], Modified Falls Efficacy Scale [14], freezing of gait [15], Rapid Eye Movement Sleep Behavior Disorder Screening Questionnaire (RBDSQ-J) [16], treatment for PD, and Hoehn and Yahr staging were reviewed.

### 2.2. The Pareidolia Test

To understand the mechanism of the pareidolias occurring in patients with PD and MSA, pareidolias were assessed using the noise pareidolia test (NPT) [6], which is a test that evokes and quantifies pareidolias and has been shown to correlate well with the occurrence of visual hallucinations. Moreover, 40 black and white images (16 × 16 cm^2^) with a spatial frequency of 1/*f*3 were used in this test. The face is included in eight. They were instructed to state whether they observed a face or not on the presentation of each image. The participants were requested to point the face when a face was observed. For each NPT image, responses were classified as [1] pareidolia (when a face was identified in the stimulus image without a face) and [2] missed (when a face was undetected in images that had a face embedded in them).

### 2.3. Statistical Analysis

All statistical analyses, except for receiver operating characteristic (ROC) curve analyses, were performed using SPSS software (version 25.0; SPSS Inc., IBM, Armonk, NY, USA). ROC curve analyses were performed using JMP Pro 14.2.0 (SAS Institute, Cary, NC, USA). Demographic data of patients with PD and MSA were compared using the *χ*^2^ test for sex; the Mann–Whitney *U* test for age at pareidolia test, disease duration, ACE-III (total score, attention/orientation, memory, language, and visuospatial), and FAB; and Student's *t*-test for ACE-III (fluency). To evaluate the differences in the pareidolia test (pareidolia and missed) between the patients with PD and those with MSA, one-way analysis of covariance was performed, with age and disease duration as covariates.

ROC curve analysis was used to evaluate the sensitivity and specificity of the pareidolia test to differentiate PD from MSA. Moreover, the optimal cutoff score was determined using the Youden method [17]. The demographic data of patients with PD with and without pareidolias were compared using the *χ*^2^ test for sex and the number of patients taking a dopamine agonist. The Mann–Whitney *U* test was performed to analyze for age during the pareidolia test, disease duration, ACE-III (total score, attention/orientation, memory, language, and visuospatial), pareidolia test (pareidolia and missed), HAMD-17, RBDSQ-J, dopamine agonist dose, catechol-*O*-methyltransferase inhibitor (COMTI), monoamine oxidase type B inhibitor (MAOBI), trihexyphenidyl, zonisamide, istradefylline, and amantadine, and Hoehn and Yahr. Student's *t*-test was used for ACE-III (fluency), STAI (state anxiety and trait anxiety), freezing of gait, levodopa dose, and levodopa equivalent units (total). Finally, Welch's test was used for FAB, whereas Fisher's exact test was performed to analyze the number of patients taking levodopa, COMTI, MAOBI, trihexyphenidyl, zonisamide, istradefylline, and amantadine and the number of deep brain stimulations postoperatively. *P* < 0.05 was considered statistically significant.

## 3. Results

Table 1 shows the demographic and clinical profiles of the subject groups included in the study. Age at the pareidolia test was significantly higher in patients with PD than in patients with MSA (*P*=0.020). Disease duration was significantly longer for patients with PD than for patients with MSA (*P* < 0.001). Except for age at the pareidolia test and disease duration, no other significant differences in the demographic characteristics among the subject groups were noted. Moreover, none of the patients met the Movement Disorder Society Task Force Level 1 criteria for PD with dementia based on the results of neuropsychological tests [18].

**Figure 1:**
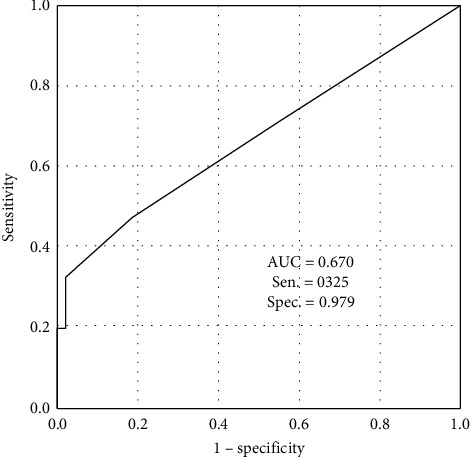
Receiver operating characteristic (ROC) curve analysis of the pareidolia test distinguishing Parkinson's disease and multiple system atrophy. AUC: area under the curve; Sen: sensitivity; Spec: specificity.

Visual hallucinations were not observed in either patients with PD or MSA. Moreover, 19 of 40 patients with PD (47.5%) showed one or more pareidolic responses: the rate was higher than patients with MSA (9 of 48 patients with MSA, 18.8%, *P*=0.004). One or more pareidolic responses were observed in 3 of 19 MSA-P patients and 6 of MSA-C patients (*P*=0.488). The number of pareidolic responses in patients with PD was significantly larger than that in patients with MSA (*P*=0.001; Table 1). The area under the curve (AUC) for the pareidolic response in the pareidolia test, which was used to differentiate patients with PD from patients with MSA, was 0.670. The optimal cutoff point for the pareidolic response in the pareidolia test was 1/2 (sensitivity = 0.325 and specificity = 0.979; Figure 1).

Patients with PD with pareidolias were defined as those showing one or more pareidolic responses in the pareidolia test. A comparison of patients with PD with and without pareidolias is shown in Table 2. Moreover, the treatment profiles of patients with PD with and without pareidolia are shown in Supplementary Table 1. STAI-state (state anxiety) score was significantly higher for patients with PD than for those without pareidolias (*P*=0.044). STAI-trait (trait anxiety) score was also significantly higher for patients with PD than for those without pareidolias (*P*=0.044). Except for the STAI score, no significant differences in the demographic characteristics and other clinical data among the two groups were noted.

## 4. Discussion

Pareidolic responses in patients with PD and MSA without hallucinations were investigated using the NPT. Pareidolic responses were more frequently found in patients with PD than in those with MSA. Although a ROC curve analysis demonstrated that the pareidolia test with the optimal cutoff score did not show high accuracy for differentiating PD from MSA, having two or more pareidolic responses in the pareidolia test was highly specific for PD. Moreover, patients with PD with the pareidolic response had higher STAI score than those without the pareidolic response, and anxiety may be associated with pareidolias in patients with PD.

Pareidolias can be found not only in patients with PD with visual hallucinations but also in those without visual hallucinations. The prevalence of pareidolias in the NPT for patients with PD without visual hallucinations was 47.5% in the current study. In a previous report including patients with PD both with and without visual hallucinations, the prevalence of pareidolias in the scene pareidolia test was 62.2%, and a subset of patients with PD without visual hallucinations exhibited pareidolias [8]. Similarly, previous studies using object identification tasks with ambiguous visual stimuli showed that a subset of patients with PD without visual hallucinations exhibited illusory misidentifications of nonexistent visual objects [19, 20]. The frequency of pareidolias detected by the pareidolia test in the present study was much higher than the prevalence of visual illusions reported in previous studies (4.2%–19%) [1, 3, 4]. The detection of visual illusions in clinical settings has to depend on self- and family reports, and obtaining reliable information when the patient does not have insight into their visual illusions or when the patient lives alone is difficult. Therefore, the underreporting of visual illusions may be associated with this discrepancy. Consistent with the results of the current study, pareidolias were observed not only in patients with DLB with visual hallucinations but also in those without visual hallucinations [5].

The pareidolia test may be a highly specific test for differentiating PD from MSA. Lewy body disease and MSA share clinical features, such as parkinsonism, autonomic failure, and cognitive impairment, and are often difficult to distinguish. Previous autopsy series including patients with antemortem MSA diagnosis showed that diagnostic accuracy was 62%–86%, and the most frequent alternative pathological diagnosis was the Lewy body disease [21–24]. Previous studies revealed that the presence of visual hallucinations was helpful in the differentiation of Lewy body disease from other non-Lewy body causes of parkinsonism including MSA [25, 26]. Pathologically confirmed patients with MSA had a low frequency of hallucinations of 5%–13%, which was shown to be significantly lower than that in Lewy body disease [23–25]. Pareidolias in patients with MSA have been rarely evaluated. Moreover, only 1 of 48 patients with MSA in the current study showed two or more pareidolic responses in the NPT. None of the nine patients with MSA reported illusion in a previous study using the Queen Square Visual Hallucination Inventory, which included a direct question for illusion [27].

Anxiety may be associated with pareidolias through developing abnormal perceptual priors/biases in Lewy body disease. The STAI has been applied in previous studies for the quantification of anxiety symptoms in patients with PD [28, 29], and its reliability and validity have been demonstrated [30, 31]. STAI-state is designed to measure a temporary state of anxiety, whereas STAI-trait measures a more enduring pattern of anxiety. In both subscales, high scores indicate high anxiety severity. In the present study, both STAI-state and STAI-trait were significantly higher in patients with PD than in those without pareidolias. Consistent with the result of the current study, a previous study in DLB showed that pareidolic illusions significantly increased under negative mood that evoked a high STAI-state score [32]. The authors of the aforementioned study suggested that the modulations of pareidolic illusions by moods were mediated by heightened abnormal perceptual bias rather than sensory deterioration based on the signal detection theory analysis [32]. In predictive coding theories, perception is not simply the passive reception of inputs from the external world but rather an active inference process based on priors and inputs [33]. Based on this view, false perception in psychosis (e.g., hallucinations and illusions) may be explained with respect to prior abnormalities [34]. A relationship between anxiety and hallucinations has been implicated in healthy people and patients with psychosis, suggesting that anxiety may be associated with false perception through fostering abnormal perceptual priors [35, 36].

This study has several limitations. First is the selection bias inherent in a retrospective study from a single tertiary center. Another potential source of selection bias in this study is that only about one-fifth of patients with PD presented to our department during the study period were included. However, as a result, this study included patients with PD with a wide range of disease duration (0.6–26.3 years) and did not include patients with overt dementia. Second, pathologic confirmation was not done, and the possibility of misdiagnosis in some cases cannot be excluded. Finally, of the two versions of the pareidolia test, the noise and scene pareidolia tests, only the NPT was used. The scene pareidolia test has been reported to be superior than the NPT in differentiating DLB from Alzheimer's disease [5]. Using the NPT rather than the scene pareidolia test in this study may have affected the diagnostic accuracy, sensitivity, and specificity. Further studies are needed to clarify which pareidolia test is more useful in discriminating PD from MSA. However, the NPT has been reported to correlate better with visual hallucinations than the scene pareidolia test [6].

## 5. Conclusions

In conclusion, pareidolias can be found in patients with PD without visual hallucinations. Moreover, the pareidolia test may be a highly specific test for differentiating PD from MSA.

## Figures and Tables

**Table 1:** Demographic and clinical profiles of the participants.

Group	PD	MSA	*P* value
Total no.	40	48	
Sex (male/female)^a^	23/17	29/19	0.782
Age at the pareidolia test, *y*, median (range)^b^	69.5 (47–82)	66 (48–82)	**0.020**
Disease duration, *y*, median (range)^b^	6.65 (0.6–26.3)	1.95 (0.7–8.6)	**<0.001**
ACE-III total score, median (range)^b^	91 (63–100)	90 (58–97)	0.491
Attention/orientation, median (range)^b^	17.5 (11–18)	17 (12–18)	0.287
Memory, median (range)^b^	23 (9–26)	22.5 (10–26)	0.787
Fluency (mean ± SD)^c^	9.8 ± 2.2	9.4 ± 2.2	0.429
Language, median (range)^b^	26 (20–26)	26 (15–26)	0.970
Visuospatial, median (range)^b^	15 (8–16)	15 (6–16)	0.325
FAB, median (range)^b^	15 (9–18)	14 (5–18)	0.102
Pareidolia test			
Pareidolia, median (range)^d^	0 (0–17)	0 (0–4)	**0.001**
Missed, median (range)^d^	0 (0-1)	0 (0-2)	0.492

PD: Parkinson's disease; MSA: multiple system atrophy; ACE-III: Addenbrooke's Cognitive Examination; SD: standard deviation; FAB: Frontal Assessment Battery. ^a^*χ*^2^ test. ^b^Mann–Whitney *U* test. ^c^Student's *t*-test. ^d^Analysis of covariance. Values in bold are statistically significant at *p* < 0.05.

**Table 2:** Demographic and clinical profiles of patients with PD with and without pareidolia.

Group	Pareidolia (+)	Pareidolia (−)	*P* value
Total no.	19	21	
Sex (male/female)^a^	11/8	12/9	0.962
Age at the pareidolia test, *y*, median (range)^b^	71 (47–79)	69 (50–82)	0.915
Disease duration, *y*, median (range)^b^	8.1 (0.8–16.8)	6.0 (0.6–26.3)	0.748
ACE-III total score, median (range)^b^	91 (63–99)	91 (76–100)	0.688
Attention/orientation, median (range)^b^	18 (11–18)	17 (14–18)	0.649
Memory, median (range)^b^	23 (13–25)	23 (9–26)	0.436
Fluency (mean ± SD)^c^	9.6 ± 2.4	9.9 ± 2.0	0.641
Language, median (range)^b^	26 (20–26)	26 (21–26)	0.520
Visuospatial, median (range)^b^	15 (8–16)	15 (9–16)	0.520
FAB, median (mean ± SD)^d^	14.4 ± 2.8	15.0 ± 1.8	0.448
Pareidolia test			
Pareidolia, median (range)^b^	3 (1–17)	0	**<0.001**
Missed, median (range)^b^	0 (0-1)	0 (0-1)	0.236
STAI			
State anxiety (mean ± SD)^c^	48.4 ± 10.5	42.5 ± 7.3	**0.044**
Trait anxiety (mean ± SD)^c^	48.8 ± 10.9	42.1 ± 9.5	**0.044**
Hamilton Rating Scale for Depression, median (range)^b^	5 (1–36)	4 (0–9)	0.093
MFES (mean ± SD)^c^	92.5 ± 38.3	107.8 ± 28.7	0.159
Freezing of gait (mean ± SD)^c^	10.5 ± 6.9	8.8 ± 5.8	0.398
RBDSQ-J, median (range)^b^	4 (1–11)	5 (0–10)	0.486
Hoehn and Yahr scale, median (range)^b^	3 (1–4)	3 (1–4)	0.789

PD: Parkinson's disease; ACE-III: Addenbrooke's Cognitive Examination; SD: standard deviation; FAB: Frontal Assessment Battery; STAI: State-Trait Anxiety Inventory; MFES: Modified Falls Efficacy Scale; RBDSQ: Rapid Eye Movement Sleep Behavior Disorder Screening Questionnaire. ^a^*χ*^2^ test. ^b^Mann–Whitney *U* test. ^c^Student's *t*-test. ^d^Welch's test. Values in bold are statistically significant at *p* < 0.05.

## Data Availability

Any data not published within the article will be anonymously shared upon request from any qualified investigator.

## Supplementary Materials

Supplementary Materials Supplementary Table 1: treatment profiles of patients with Parkinson's disease with and without pareidolia.
