# Supplementary material for: Pareidolia in Parkinson's Disease and Multiple System Atrophy
Source: Parkinsons Dis. 2021 Oct 31;2021:2704755. doi: 10.1155/2021/2704755 (PMC8572613; doi:10.1155/2021/2704755)
Supplement: Supplementary Materials — Supplementary Table 1: treatment profiles of patients with Parkinson's disease with and without pareidolia. [file 2704755.f1.docx]

| Supplementary Table 1. Treatment profiles of patients with Parkinson's disease with and without pareidolia. | | | | |
| --- | --- | --- | --- | --- |
| Group | | Pareidolia (+) | Pareidolia (−) | *P* value |
| Total no. | | 19 | 21 |  |
|  | Dopamine agonist (+/−)^a^ | 9/10 | 11/10 | 0.752 |
|  | Dopamine agonist (LEU), mg, median (range)^b^ | 0 (0–682) | 33.0 (0–545) | 0.748 |
|  | Levodopa (+/−)^c^ | 18/1 | 18/3 | 0.342 |
|  | Levodopa, mg, (mean ± SD)^d^ | 357.9 ± 184.3 | 307.1 ± 205.1 | 0.417 |
|  | COMTI (+/−)^c^ | 0/19 | 2/19 | 0.269 |
|  | COMTI (LEU), mg, median (range)^b^ | 0 | 0 (0–148.5) | 0.611 |
|  | MAOBI (+/−)^c^ | 4/15 | 6/15 | 0.429 |
|  | MAOBI (LEU), mg, median (range)^b^ | 0 (0–100) | 0 (0–50) | 0.768 |
|  | Trihexyphenidyl (+/−)^c^ | 1/18 | 1/20 | 0.731 |
|  | Trihexyphenidyl, mg, median (range)^b^ | 0 (0–2) | 0 (0–2) | 0.979 |
|  | Zonisamide (+/−)^c^ | 3/16 | 2/19 | 0.451 |
|  | Zonisamide, mg, median (range)^b^ | 0 (0–50) | 0 (0–25) | 0.728 |
|  | Istradefyline (+/−)^c^ | 2/17 | 6/15 | 0.152 |
|  | Istradefyline, mg, median (range)^b^ | 0 (0–40) | 0 (0–40) | 0.347 |
|  | Amantadine (+/−)^c^ | 4/15 | 5/16 | 0.569 |
|  | Amantadine (LEU), mg, median (range)^b^ | 0 (0–300) | 0 (0–300) | 0.872 |
|  | LEU (total), (mean ± SD)^d^ | 515 ± 413.4 | 516.4 ± 374.9 | 0.991 |
|  | DBS (+/−)^c^ | 0/19 | 3/18 | 0.135 |
| Hoehn & Yahr, median (range)^b^ | | 3 (1–4) | 3 (1–4) | 0.789 |
| PD, Parkinson's disease; SD, standard deviation; LEU, Levodopa equivalent units; COMTI, catechol-*O*-methyltransferase inhibitors; MAOBI, monoamine oxidase type B inhibitors; DBS, deep brain stimulation ^a^*χ*2 test ^b^Mann–Whitney *U* test ^c^Fisher's exact test  ^d^Student's *t*-test | | | | |
